# A human obesity-associated MC4R mutation with defective G_q/11_**α** signaling leads to hyperphagia in mice

**DOI:** 10.1172/JCI165418

**Published:** 2024-01-04

**Authors:** Peter J. Metzger, Aileen Zhang, Bradley A. Carlson, Hui Sun, Zhenzhong Cui, Yongqi Li, Marshal T. Jahnke, Daniel R. Layton, Meenakshi B. Gupta, Naili Liu, Evi Kostenis, Oksana Gavrilova, Min Chen, Lee S. Weinstein

**Affiliations:** 1 Metabolic Diseases Branch and; 2 Mouse Metabolism Core Laboratory, National Institute of Diabetes and Digestive and Kidney Diseases (NIDDK), NIH, Bethesda, Maryland, USA.; 3 Molecular, Cellular, and Pharmacobiology Section, Institute for Pharmaceutical Biology, University of Bonn, Bonn, Germany.

**Keywords:** Metabolism, G proteins, Melanocortin, Signal transduction

## Abstract

Melanocortin 4 receptor (MC4R) mutations are the most common cause of human monogenic obesity and are associated with hyperphagia and increased linear growth. While MC4R is known to activate G_s_α/cAMP signaling, a substantial proportion of obesity-associated MC4R mutations do not affect MC4R/G_s_α signaling. To further explore the role of specific MC4R signaling pathways in the regulation of energy balance, we examined the signaling properties of one such mutant, MC4R (F51L), as well as the metabolic consequences of MC4RF51L mutation in mice. The MC4RF51L mutation produced a specific defect in MC4R/G_q/11_α signaling and led to obesity, hyperphagia, and increased linear growth in mice. The ability of a melanocortin agonist to acutely inhibit food intake when delivered to the paraventricular nucleus (PVN) was lost in MC4RF51L mice, as well as in WT mice in which a specific G_q/11_α inhibitor was delivered to the PVN; this provided evidence that a G_s_α-independent signaling pathway, namely G_q/11_α, significantly contributes to the actions of MC4R on food intake and linear growth. These results suggest that a biased MC4R agonist that primarily activates G_q/11_α may be a potential agent to treat obesity with limited untoward cardiovascular and other side effects.

## Abstract 2

<p>A melanocortin 4 receptor mutation associated with human obesity leads to hyperphagia and obesity in mice and has a specific defect in Gq/11 activation.</p>

## Introduction

Melanocortins are neurotransmitters that act in the CNS to promote negative energy balance via reduced food intake and increased energy expenditure and sympathetic nervous system (SNS) activity. Melanocortin 4 receptor (MC4R) is the main receptor that mediates the effects of melanocortins on food intake and energy expenditure. MC4R is an important regulator of energy balance in humans, as loss-of-function MC4R mutations constitute the most common form of monogenic obesity (1); and more than 150 pathogenic MC4R variants have been identified in patients with severe, early-onset obesity accompanied by hyperphagia, increased linear growth, impaired glucose metabolism (2) and decreased heart rate and blood pressure compared with similarly obese controls (3). MC4R deficiency also causes obesity associated with increased food intake and reduced energy expenditure in mice (4, 5). MC4R agonism is a logical therapeutic target for the treatment of obesity. However, the use of MC4R agonists has been limited by untoward cardiovascular side effects (3).

While MC4R expression in the CNS is fairly widespread (6, 7), a major site of MC4R action affecting acute inhibition of food intake (8–10) is the paraventricular nucleus of the hypothalamus (PVN), where it is activated by melanocortins released from proopiomelanocortin (POMC) neurons originating from the arcuate nucleus of the hypothalamus (ARC). PVN-specific reexpression of MC4R in MC4R-null mice reverses hyperphagia but does not correct the impaired energy expenditure (8), consistent with MC4R’s effects on food intake occurring primarily in the PVN and its effects on energy expenditure occurring at other CNS sites. MC4R activation leads to induction of the transcription factor Sim1 (11), and mutations in *SIM1* and *Sim1* lead to obesity, hyperphagia, and increased linear growth in humans and mice, respectively (12–15).

It is well established that activated MC4Rs stimulate the G protein G_s_α, which stimulates adenylyl cyclase to increase intracellular cAMP accumulation. Heterozygous G_s_α-inactivating mutations lead to obesity in patients with pseudohypoparathyroidism type 1A (PHP1A) (16) and in mice (17), but only when the mutation is on the maternal allele due to tissue-specific imprinting of *GNAS/Gnas*, the gene encoding G_s_α. Disruption of the maternal G_s_α allele limited to the CNS (mBrGsKO) produces the same obesity phenotype, which is associated with reduced energy expenditure, impaired cold-induced thermogenesis, insulin resistance, and glucose intolerance (18). However, unlike what is observed with loss of MC4R (1, 4, 19), obesity in mBrGsKO mice is not driven by hyperphagia, and the anorectic effects of a melanocortin agonist remain intact (18). Moreover, unlike Mc4r-null mice, mBrGsKO mice do not show increased linear growth (18). It appears that obesity in mBrGsKO mice is mediated by G_s_α signaling defects in the dorsomedial hypothalamus (DMH), leading to reduced energy expenditure (20). Mice with G_s_α deficiency limited to the PVN (mPVNGsKO) also do not show hyperphagia or an impairment in melanocortin-induced food intake inhibition (21).

The results from these mouse studies suggest that the effects of MC4R action in the PVN on food intake and linear growth are mediated by downstream signaling pathways that are independent of G_s_α, which is further supported by studies showing that a significant proportion of human MC4R mutations associated with monogenic obesity do not show significant defects in activation of G_s_α/cAMP signaling (22–24). One study correlating signaling properties of naturally occurring MC4R variants with BMI concluded that differences in MC4R/G_s_α signaling do not account for most of the variance in BMI (25). Several alternate pathways downstream of MC4R have been implicated in mediating its effects on food intake and body weight, including β-arrestin recruitment (25, 26), closure of the inward rectifying potassium channel Kir7.1 (27), and activation of MAPK (28, 29), although there is no direct evidence that strongly links these pathways in the PVN to the regulation of energy balance. We have shown that PVN-specific deletion of both G_q_α and G_11_α (PVNGq/11KO), 2 homologous and ubiquitously expressed G protein α-subunits that couple receptors to PLC, recapitulates the effects of MC4R deficiency on food intake and linear growth — namely, severe obesity associated with hyperphagia and loss of the anorectic response to melanocortin agonist, along with increased linear growth (11). Moreover, some MC4R mutants with normal G_s_α signaling have been demonstrated to have impaired PLC activation as measured by a nuclear factor of activated T cells (NFAT) reporter system (30).

To further explore the role of MC4R signaling pathways in the regulation of energy balance, we examined in detail the physiological consequences in mice and signaling properties of one mutant MC4R that is associated with early-onset obesity in humans and has been reported to have relatively intact G_s_α signaling (MC4RF51L, phenylalanine 51 to leucine) (23). Our results showed that this mutation produced a specific defect in MC4R/G_q/11_α signaling and that the mutation in mice led to a phenotype closely related to that seen in PVNGq/11KO mice, providing evidence that G_q/11_α signaling, rather than G_s_α signaling, mediates the actions of MC4R on food intake and linear growth. This conclusion is further supported by the observation that a specific G_q/11_α inhibitor delivered to the PVN of WT mice blocked the ability of a melanocortin agonist to acutely inhibit food intake. Biased MC4R agonists that primarily activate G_q/11_α signaling may lead to food intake suppression and weight loss with limited untoward limited effects.

## Results

### MC4RF51L mice develop obesity without disruption of MC4R/G_s_α/cAMP signaling.

**Figure 1:**
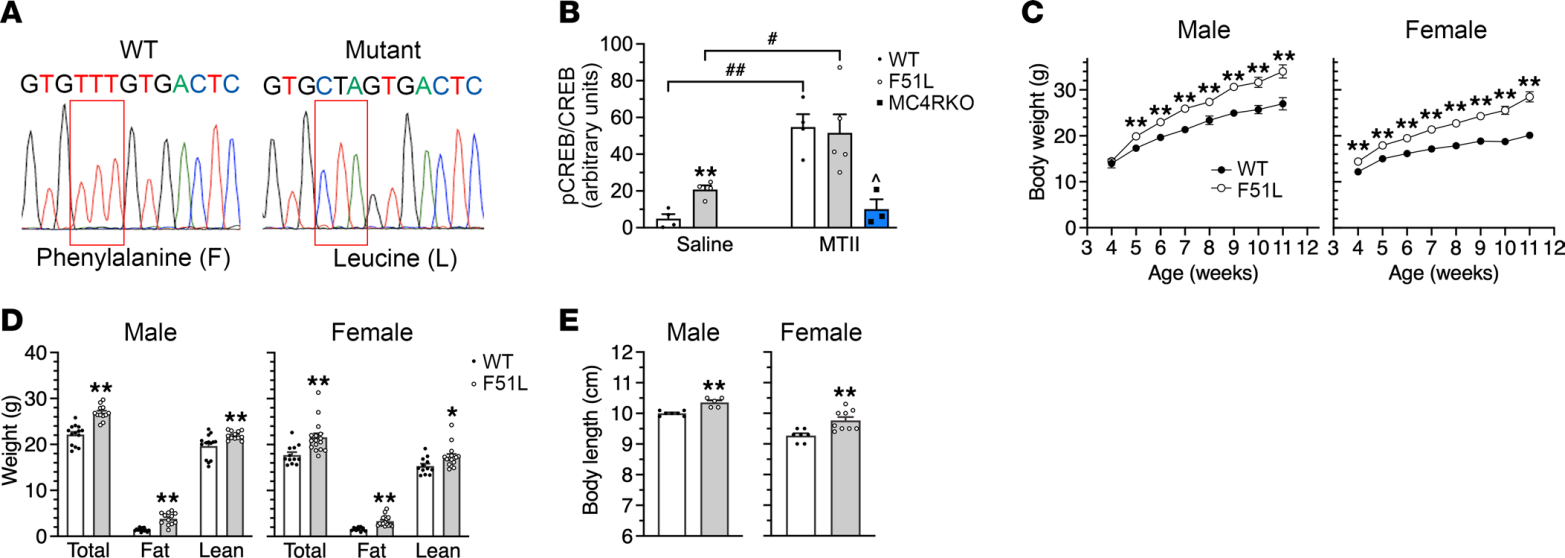
MC4RF51L mice develop obesity without disruption of MC4R/G_s_α signaling. (**A**) DNA sequencing showing conversion of phenylalanine 51 (TTT) in WT mice to leucine (CTA) in MC4RF51L mice. (**B**) Quantification of pCREB/total CREB in PVN of WT, MC4RF51L (F51L), and MC4RKO mice after i.p. injection of saline or MTII (*n* = 3–5/group; ^#^*P* < 0.05, ^##^*P* < 0.01 vs. saline; ^*P* < 0.05 vs. WT and MC4RF51L mice). (**C**) Body weights of male (left) and female (right) MC4RF51L and WT mice measured weekly from week 4 to 11 (males, *n* = 19–24/group; females, *n* = 20–24/group). (**D**) Total body, fat and lean mass of 8-week-old male (*n* = 12–14/group) and female (*n* = 12–16/group) MC4RF51L and WT mice. (**E**) Body length of 8-week-old male and female of MC4RF51L and WT mice (males, *n* = 5–7/group; females, *n* = 7–9/group). Data represent mean ± SEM. *P < 0.05, **P < 0.01 vs. WT by unpaired *t* test or 2-way ANOVA.

MC4RF51L mice were generated by CRISPR/Cas9 to direct an MC4R site mutation at amino acid position 51 with phenylalanine replaced by leucine (Figure 1A) and mating to homozygosity. The human and mouse MC4R protein sequences are highly homologous (94% identical, 97% conserved), and the F51 residue is in a highly conserved region (14 amino acids upstream through 112 downstream of F51 are identical between the species).

To verify that MC4R/G_s_α/cAMP signaling was maintained in MC4RF51L mice in vivo, we administered the MC4R agonist melanotan II (MTII; 10 μg/g body weight) i.p. to mice and measured phosphorylation levels of CREB (pCREB) in the PVN by immunofluorescence; this served as a readout of cAMP production, as cAMP stimulates the phosphorylation of CREB via activation of PKA (Figure 1B and Supplemental Figure 1; supplemental material available online with this article; https://doi.org/10.1172/JCI165418DS1). MC4RF51L mice and WT littermates had similar levels of total CREB protein. After administration of saline, pCREB signals were low in WT PVN, while there were significantly higher levels of pCREB in the PVN of MC4RF51L mice. This was likely due to the fact that MC4RF51L mice had increased leptinergic signaling (see Table 1), leading to greater melanocortinergic input to the PVN, which would be expected to increase CREB phosphorylation if MC4R/G_s_α/cAMP signaling is intact in the mutant receptor — although other mechanisms associated with obesity may also be involved. After MTII administration, CREB phosphorylation significantly increased to similar levels in WT and MC4RF51L PVN. Concomitant administration of MTII into MC4RKO mice resulted in low pCREB levels in the PVN that were similar to those seen in WT after saline administration and significantly lower than those seen in MTII-treated WT or MC4RF51L mice, indicating that the observed pCREB responses to MTII in WT and MC4RF51L mice were primarily due to signaling through MC4R, as opposed to MC3R or other members of the melanocortin receptor family. Overall these results are consistent with the mutant MC4R receptor having intact downstream G_s_α/cAMP signaling in the PVN, as was observed in the present study and by others (23) in cell-based signaling assays.

MC4RF51L mice appeared normal at birth but rapidly gained more weight than WT littermates (Figure 1C). Male mutants had significantly increased body weight at 5 weeks of age and were 21% heavier than their WT littermates at 11 weeks of age, while female mutants became significantly heavier starting at 4 weeks and gained 29% more weight than their WT littermates by 11 weeks of age. The weight gain in MC4RF51L mice was attributed to significant 2- to 3-fold increases in fat mass and small, but significant, increases in lean mass (Figure 1D). At least some of the increase in lean mass was attributable to an increase in body length (Figure 1E), a finding that was also observed in mice with germline MC4R mutations (4) and mice with PVN-specific G_q/11_α deficiency (11) but not in obese mBrGsKO mice (18). Consistent with increased adiposity, MC4RF51L mice developed severe hyperleptinemia, with circulating leptin levels more than 20-fold higher in MC4RF51L mice compared with their WT littermates at 12 weeks of age (Table 1).

### Obesity in MC4RF51L mice is associated with hyperphagia.

**Figure 2:**
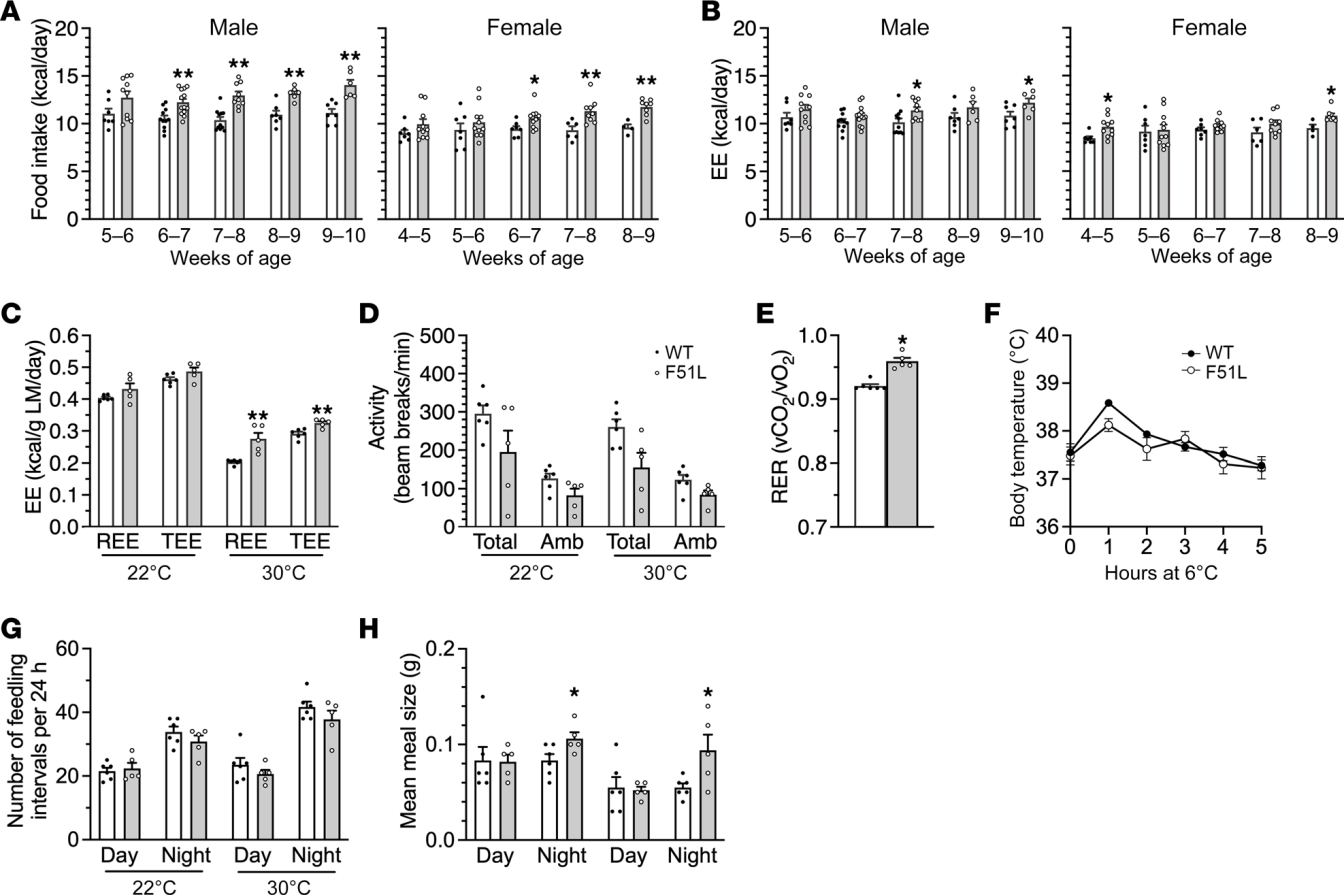
Obesity in MC4RF51L mice is associated with hyperphagia. (**A**) Average daily food intake measured every 7 days for 5 weeks in male and female MC4RF51L and WT mice (*n* = 4–13/group). (**B**) Average daily energy expenditure (EE) calculated weekly for 5 weeks in male and female MC4RF51L and WT mice. (**C**) REE and TEE measured by indirect calorimetry and normalized to lean mass (LM) in 3-month-old male MC4RF51L and WT mice (*n* = 5–6/group). (**D**) Total and ambulatory (Amb) activity levels in male MC4RF51L and WT mice at 3 months of age (*n* = 5–6/group). (**E**) RERs (vCO_2_/vO_2_) at 22°C in 3-month-old male MC4RF51L and WT mice at 3 months of age (*n* = 5–6/group). (**F**) Cold tolerance test. Rectal temperature in 4-month-old male MC4RF51L and WT mice at room temperature (0 hours) or at the indicated time points after being placed in an environment with a 6°C ambient temperature (*n* = 6–9/group). Meal frequency (**G**) and meal size (**H**) measured in 3 month-old male mice during daylight and nighttime hours at 22°C and 30°C (*n* = 5–6/group). Meal size was significantly increased in MC4RF51L mice compared with WT mice as determined by 2-way ANOVA. Data represent mean ± SEM. *P < 0.05, **P < 0.01 vs. WT by unpaired *t* test.

To determine the extent that differences in food intake and energy expenditure contribute to the increased weight gain in MC4RF51L mice, we measured food consumption and body composition every 7 days over a 5-week period in males starting at 5 weeks and in females starting at 4 weeks of age, and calculated energy intake and energy expenditure based on these measurements (31). During this period, both male and female MC4RF51L mice ate significantly more than their WT littermates (Figure 2A). On the other hand, energy expenditure was not decreased and in fact was increased in both males and females at various time points, reflecting their increased body mass (Figure 2B).

We also studied parameters of energy metabolism in 3-month-old male mice using an Oxymax–Comprehensive Lab Animal Monitoring System (CLAMS, Columbus Instruments). indirect calorimetry showed no differences in resting (REE) and total energy expenditure (TEE) between the genotypes at room temperature (22°C) when normalized to lean mass (Figure 2C). The same measurements performed at thermoneutrality (30°C), a condition in which SNS activity and thermogenesis are minimized, showed increased REE and TEE in MC4RF51L mice when normalized to lean mass (Figure 2C). Physical activity tended to be lower in MC4RF51L mice than in WT mice, but these differences did not reach statistical significance (Figure 2D). Respiratory exchange ratio (RER; vCO_2_/vO_2_) was increased in MC4RF51L mice (Figure 2E), suggesting greater utilization of carbohydrates relative to fats as a fuel source in the mutant mice. Examination of food consumption patterns revealed no differences in feeding frequency between WT and MC4RF51L mice during the day or night, whether at 22°C or 30°C (Figure 2G). However, meal size was significantly larger in MC4RF51L mice as compared with WT during the night at both 22°C and 30°C (Figure 2H), consistent with a higher satiety threshold in MC4RF51L mice. These findings are consistent with previous studies showing that melanocortins working through MC4R receptors primarily affect meal size, rather than meal frequency (32–34).

MC4RF51L mice did not show evidence of acute cold intolerance, as they were able to maintain their body temperature over 5 hours at 6°C (Figure 2F), consistent with there being no overt defect in cold-induced thermogenesis, which we had previously observed in mice with whole-brain G_s_α deficiency but not in mice with G_s_α deficiency limited to the PVN (21). In summary, obesity in MC4RF51L mice was primarily associated with hyperphagia as a result of increased meal size. On the other hand, MC4RF51L mice showed no evidence of reduced energy expenditure or impaired cold-induced thermogenesis, which are both features of MC4R-null mice (4, 5, 8).

### Melanocortin-mediated food intake inhibition is impaired in MC4RF51L mice.

**Figure 3:**
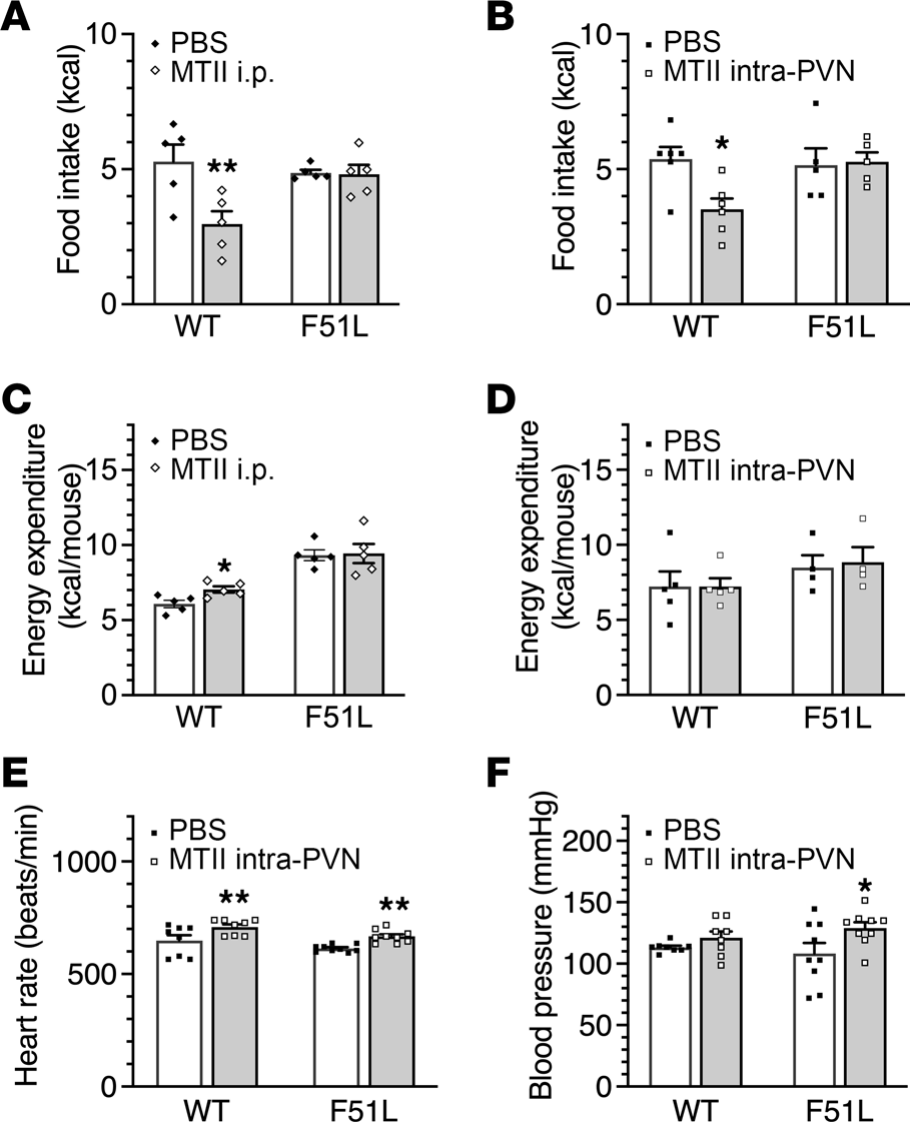
Food intake, energy expenditure, heart rate, and blood pressure responses to MTII. Food intake following i.p. injection of PBS or MTII in 3-month-old male MC4RF51L and WT mice (*n* = 5/group) (**A**) or intra-PVN injection of PBS or MTII in 4- to 5-month-old male MC4RF51L and WT mice (*n* = 5–6/group) (**B**). Energy expenditure following i.p. injection of PBS or MTII in 3-month-old male MC4RF51L and WT mice (*n* = 5–6/group) (**C**) or intra-PVN injection of PBS or MTII in 4- to 5-month-old male MC4RF51L and WT mice (*n* = 4–5/group) (**D**). Heart rate (**E**) and mean blood pressure (**F**) after intra-PVN injection of PBS or MTII in 3-month-old female MC4RF51L and WT mice (*n* = 8–9/group). Data represent mean ± SEM. *P < 0.05, **P < 0.01 vs. WT by 2-way ANOVA.

We have previously shown that within the PVN, MC4R-mediated food intake inhibition appears to be mediated by G_q/11_α, while MC4R-mediated cardiovascular effects are mediated by G_s_α (11). To examine the effects of the MC4RF51L mutation on physiological responses to melanocortins, we delivered the melanocortin agonist MTII either systemically (i.p.) or directly into the PVN via cannula in MC4RF51L and WT mice and examined acute food intake, energy expenditure, and cardiovascular responses. While either i.p. (Figure 3A) or intra-PVN injection of MTII (Figure 3B) led to reduced food intake in WT mice, these effects on food intake were absent in MC4RF51L mice. While systemic MTII resulted in a small, but significant, increase in energy expenditure in WT mice, no increase in energy expenditure was observed in MC4RF51L mice (Figure 3C). Presumably this defect in melanocortin-stimulated energy expenditure was not occurring at the PVN, as direct delivery of MTII to the PVN had no effect on energy expenditure in WT or MC4RF51L mice (Figure 3D), consistent with prior studies showing that melanocortin actions on energy expenditure are not mediated within the PVN (8). In contrast to what we observed for food intake, MC4RF51L mice had normal heart rate and blood pressure at baseline, and the responses of both heart rate and blood pressure to intra-PVN delivery of MTII remained intact (Figure 3, E and F). Overall, the food intake and cardiovascular responses of MC4RF51L mice to intra-PVN MTII were similar to what was observed in mice with PVN-specific G_q/11_α deficiency (11) and were consistent with MC4RF51L mice having a specific defect in MC4R/G_q/11_α signaling.

### Glucose and lipid metabolism in MC4RF51L mice.

**Figure 4:**
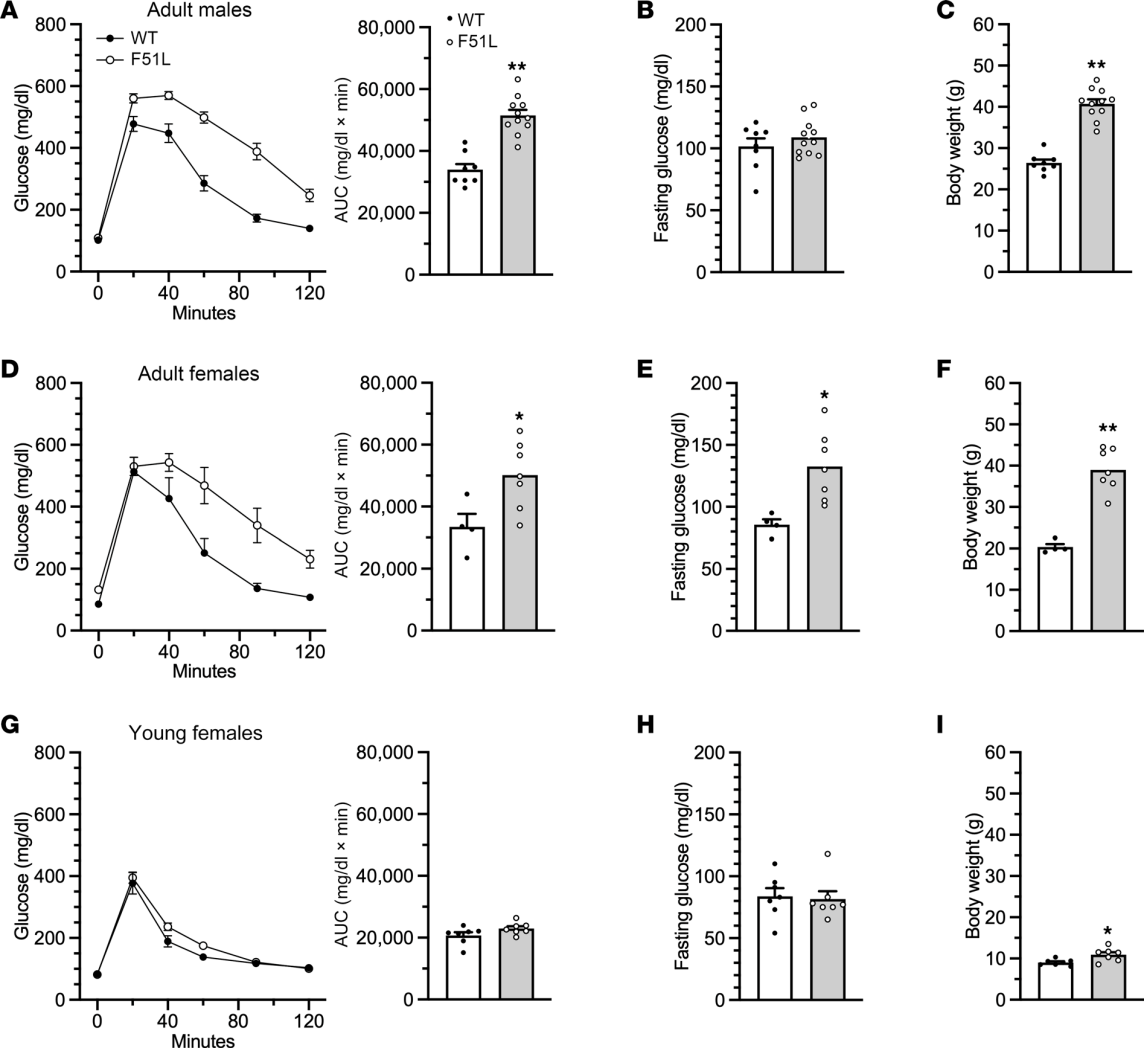
Glucose metabolism in MC4RF51L mice. (**A**–**C**) Glucose metabolism in 6- to 7-month-old male MC4RF51L and WT mice (*n* = 8–11/group). Glucose tolerance test with AUC shown on the right (**A**), fasting glucose levels (**B**), and body weights at the time of glucose tolerance tests (**C**). (**D**–**F**) Glucose metabolism in 6- to 7-month-old female MC4RF51L and WT mice (*n* = 4–7/group). Glucose tolerance test with AUC shown to the right (**D**), fasting glucose levels (**E**), and body weights at the time of glucose tolerance tests (**F**). (**G**–**I**) Glucose metabolism in 4- to 5-week-old female MC4RF51L and WT mice (*n* = 7/group). Glucose tolerance test with AUC shown on the right (**G**), fasting glucose levels, (**H**) and body weights at the time of glucose tolerance tests (**I**). Data represent mean ± SEM. *P < 0.05, **P < 0.01 vs. WT by unpaired *t* tests.

Adult MC4RF51L mice showed impaired glucose metabolism, with severe glucose intolerance in both male (Figure 4A) and female (Figure 4D) MC4RF51L mice at 6–7 months of age, after the establishment of obesity (Figure 4, C and F). Fasting glucose levels were unaffected in male MC4RF51L mice (Figure 4B) but significantly increased in female MC4RF51L mice (Figure 4E). Serum measurements in 3-month-old female mice in the randomly fed state showed significantly elevated insulin levels in MC4RF51L mice, with glucose levels remaining unaffected (Table 1). To determine whether abnormal glucose metabolism is independent of obesity, we assessed glucose homeostasis in young female MC4RF51L and WT mice at 4–5 weeks of age. Although body weight was only slightly, though significantly, increased in MC4RF51L mice at this age (Figure 4I), both glucose tolerance (Figure 4G) and fasting glucose (Figure 4H) were similar in MC4RF51L and WT mice. These results indicate that abnormal glucose homeostasis in MC4RF51L mice was secondary to obesity rather than to a primary defect in glucose metabolism, despite the fact that prior studies have shown that the loss of MC4R (35, 36) or G_s_α in the CNS (18) leads to a primary defect in glucose metabolism. Adult female MC4RF51L mice had significantly higher serum levels of cholesterol, triglycerides, and free fatty acids in the fed state, while adiponectin levels were unaffected (Table 1). It is unclear to what extent hyperlipidemia in MC4RF51L mice results from obesity versus impaired melanocortin action, as melanocortins within the CNS have been shown to directly regulate lipid metabolism (37).

### Hyperphagia significantly contributes to the metabolic phenotype of MC4RF51L mice.

**Figure 5:**
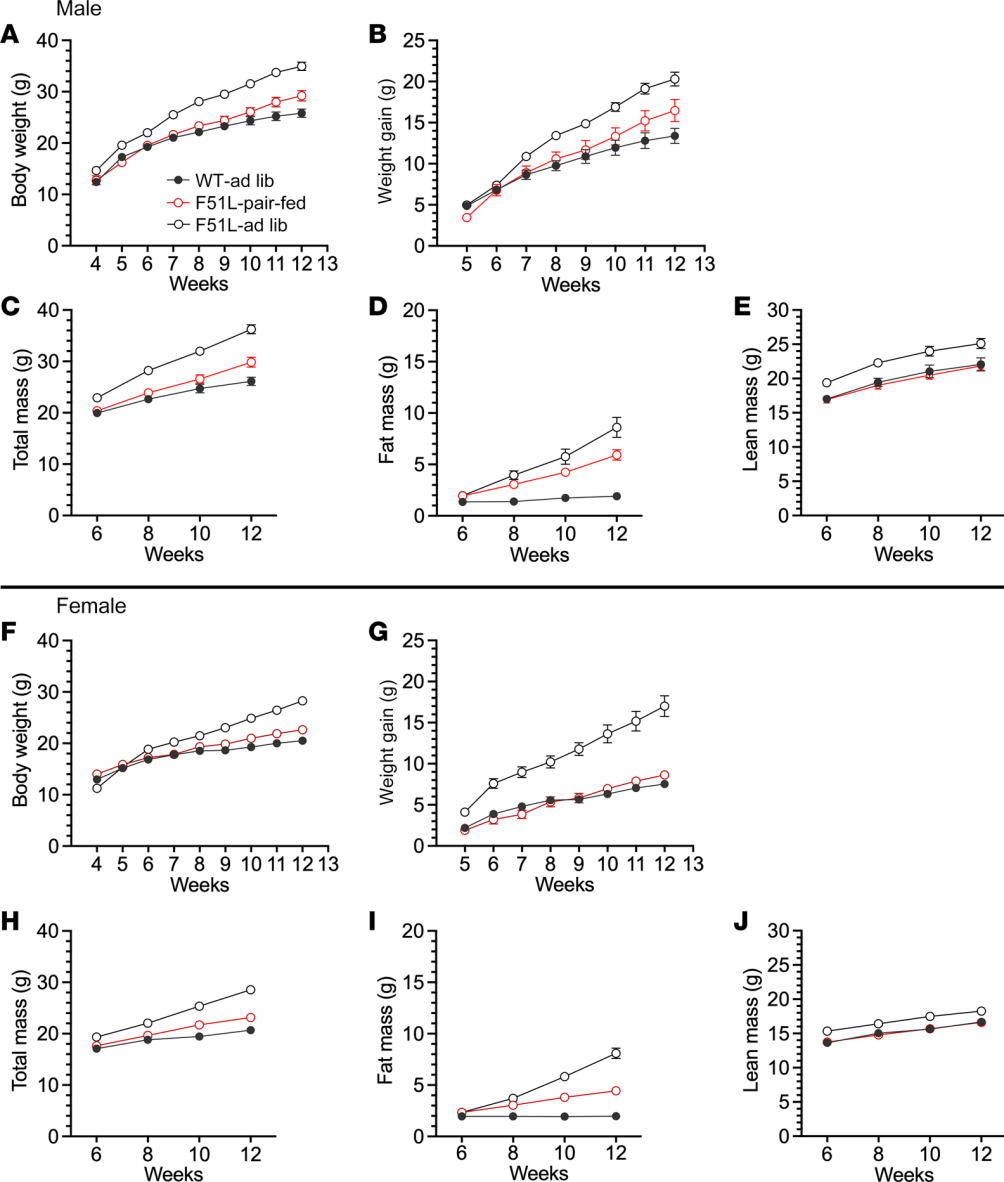
Effect of pair feeding on body weight and composition. Body weight (**A**) and weight gain (**B**) during an 8 week period (weeks 4–12) in male ad libitum fed WT (WT-ad lib), ad-libitum fed MC4RF51L mice (F51L-ad lib) and MC4RF51L mice pair-fed to WT (F51L-pair-fed). (**C**–**E**) Body composition in male mice measured at weeks 6, 8, 10, and 12 during the pair-feeding study showing total body mass (**C**), fat mass (**D**), and lean mass (**E**) (*n* = 10–12/group). Body weight (**F**) and weight gain (**G**) during pair-feeding experiment in females. (**H**–**J**) Body composition showing total body mass (**H**), fat mass (**I**), and lean mass (**J**) in females (*n* = 9–16/group). Data represent mean ± SEM. Based on 2-way ANOVA, data for all 3 groups are statistically significantly different from each other in all panels except **E**, **G**, and **H**, in which results for ad libitum fed MC4RF51L mice are significantly different from those of the other 2 groups.

**Figure 6:**
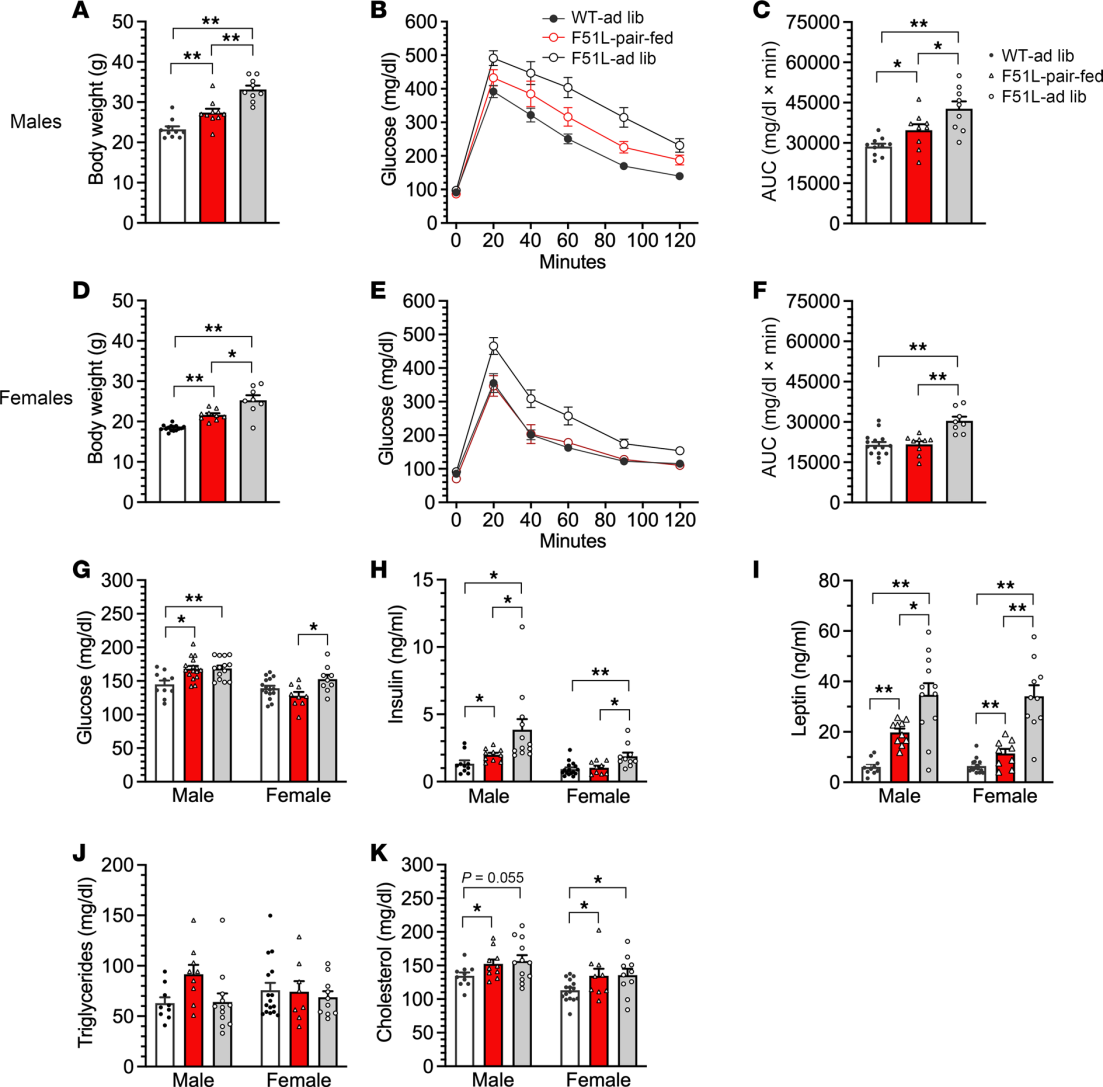
Glucose and lipid metabolism after pair feeding. Body weight (**A**), glucose tolerance (**B**), and glucose tolerance AUC (**C**) at the end of the pair-feeding study (week 12) in male WT-ad-lib, MC4RF51L-ad-lib, and MC4RF51L pair-fed mice (*n* = 9–10/group). Body weight (**D**), glucose tolerance (**E**), and glucose tolerance AUC (**F**) at the end of the pair-feeding study in female mice (*n* = 8–15/group). Nonfasted serum glucose (**G**; *n* = 10–16/group for males and 9–16/group for females), insulin (**H**; *n* = 10–12/group for males and 9–16/group for females), leptin (**I**; *n* = 10–12/group for males and 9–16/group for females), triglycerides (**J**; *n* = 9–12/group for males and 8–16/group for females), and total cholesterol (**K**; *n* = 10–12/group for males and 9–16/group for females) at the end of the pair-feeding study. Data represent mean ± SEM. *P < 0.05, **P < 0.01 by 1-way ANOVA.

In order to examine the extent to which the metabolic phenotype of MC4RF51L mice is accounted for by excess energy intake, we performed a pair-feeding experiment in which a group of MC4RF51L mice were provided an amount of food identical to that of paired WT mice on an ad libitum diet for an 8-week period from the start of week 4 until the start of week 12 after birth. Another group of MC4RF51L mice were fed ad libitum simultaneously. Body weight curves in males showed that pair-fed MC4RF51L mice gained slightly, but significantly, more weight than WT mice, although the weight gain was significantly lower than that observed in ad libitum fed MC4RF51L mice (Figure 5, A and B). Body composition confirmed that the excess weight gain in pair-fed mice was accounted for by an increase in fat mass (Figure 5, C–E). Consistent with these findings, pair-fed MC4RF51L mice had leptin levels at the end of the study that were significantly higher than those of WT mice but significantly lower than those of ad libitum fed MC4RF51L mice (Figure 6I). The findings were similar for female mice, although the increases in body weight gain, fat mass gain, and serum leptin levels in pair-fed MC4RF51L relative to WT mice were more subtle than those observed in males (Figure 5, F–J, and Figure 6I). Energy balance studies performed during the experiments confirmed once again that both male and female ad libitum fed MC4RF51L mice ate significantly more than WT mice (Supplemental Figure 2, A and C). Reduced food intake in pair-fed MC4RF51L mice led to reduced energy expenditure compared with ad libitum fed MC4RF51L mice (Supplemental Figure 2, B and D). Overall, these results confirm that hyperphagia significantly contributes to the obesity seen in MC4RF51L mice. However, pair-fed MC4RF51L mice (particularly males) still gained more fat mass compared with WT mice with similar energy intake.

Examination of glucose metabolism at the end of the pair-feeding study revealed evidence that male pair-fed MC4RF51L mice — with mean body weights that were higher than those of WT but lower than those of ad libitum fed MC4RF51L mice (Figure 6A) — had impaired glucose metabolism with reduced glucose tolerance (Figure 6, B and C) and increased glucose and insulin levels (Figure 6, G and H) compared with WT mice; however, these parameters (except for random serum glucose levels) were significantly improved compared with ad libitum fed MC4RF51L mice. In contrast, female pair-fed MC4RF51L mice, which also had a significant increase in body weight compared with WT mice (Figure 6D), showed no differences in glucose tolerance or random glucose or insulin levels compared with WT mice, while ad libitum fed MC4RF51L mice showed significant glucose intolerance and elevations in serum glucose and insulin levels (Figure 6, E–H). There were no differences in triglyceride levels between groups (Figure 6J), while pair-fed and ad libitum fed MC4RF51L mice had similarly elevated total cholesterol levels compared with WT mice (Figure 6K). Overall, these results confirm that impaired glucose metabolism in female MC4RF51L mice was completely accounted for by increased energy intake and obesity. In male mice, the partial impairment in glucose metabolism that remains after pair feeding may be secondary to the fact that these mice still have increased adiposity.

### MC4RF51L has a specific defect in G_q/11_α signaling.

**Figure 7:**
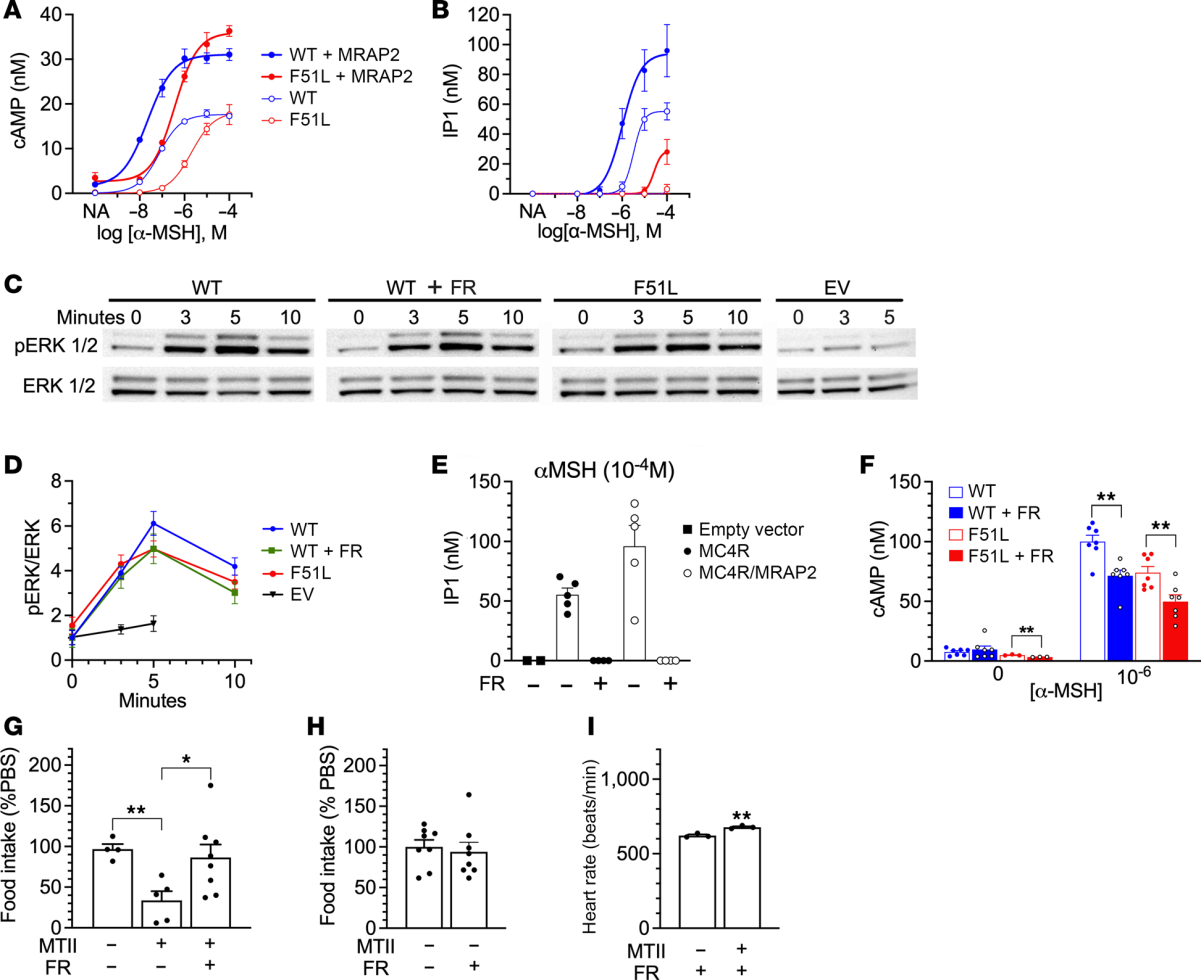
MC4RF51L has impaired G_q/11_α signaling. Dose response curves for cAMP (*n* = 3) (**A**) and IP1 (*n* = 5) (**B**) in parental HEK293 or HEK293^MRAP2^ cells that were transfected with WT or mutant (F51L) MC4R plasmids. (**C** and **D**) Representative experiment showing immunoblots probed with antibodies for phospho-ERK1/2 (p-ERK; top row) and total ERK1/2 (bottom row) in lysates from HEK293^MRAP2^ cells transfected with WT, mutant (F51L) MC4R or empty vector (EV), pretreated with the G_q/11_α inhibitor FR900359 (FR) or vehicle and incubated with 10^–5^ M α-MSH at the indicated times in minutes (**C**), with quantification of pERK/total ERK ratios normalized to WT at time 0 (*n* = 8–9/group; except for EV, *n* = 3–5/group) (**D**). (**E**) IP1 generation in response to 10^–4^ M α-MSH in HEK293 cells transfected with empty vector or HEK293 or HEK293^MRAP2^ cells transfected with WT MC4R after pretreatment with either FR or vehicle (*n* = 2–6/group). (**F**) cAMP generation in response to 0 or 10^–6^ M α-MSH in HEK293^MRAP2^ cells transfected with WT or mutant MC4R that were pretreated with either FR or vehicle (*n* = 3–7/group). (**G**) Food intake (normalized to PBS injection) in response to intra-PVN administration of vehicle alone, MTII, or MTII + FR in 4- to 6-month-old male WT mice (*n* = 4–8/group). (**H**) Food intake (normalized to PBS injection) in response to intra-PVN administration of FR alone (*n* = 8/group). (**I**) Effect of intra-PVN FR injection on heart rate response to intra-PVN MTII injection (*n* = 3/group). Data represent mean ± SEM. *P < 0.05, **P < 0.01 by unpaired *t* test or 1-way ANOVA.

Melanocortin receptor accessory protein 2 (MRAP2) is expressed in the hypothalamus, interacts with MC4R, and is required for normal energy balance and MC4R signaling (38–41). We therefore generated a cell line in which MRAP2 was stably transfected into HEK293 cells (HEK293^MRAP2^) to examine the signaling properties of WT MC4R and MC4RF51L. MRAP2 expression in HEK293^MRAP2^ cells and its absence in parental HEK293 cells was confirmed by both quantitative real-time PCR (qRT-PCR) and immunoblotting (Supplemental Figure 3). We first examined the ability of α-melanocyte–stimulating hormone (α-MSH) to stimulate cAMP accumulation in parental HEK293 or HEK293^MRAP2^ cells that were transiently transfected with either WT MC4R or MC4RF51L plasmid in the presence of the phosphodiesterase inhibitor IBMX. For both WT and mutant MC4R, the maximal response was doubled and the EC_50_ decreased by at least 1 order of magnitude in the presence of MRAP2 (Figure 7A), indicating that MRAP2 is required for optimal MC4R/G_s_α/cAMP signaling. Maximal cAMP accumulation was similar in cells transfected with WT MC4R or MC4RF51L, in both the presence and absence of MRAP2 (Figure 7A). This finding was consistent with prior results (22, 23) and our observation that CREB phosphorylation in response to MTII was unaffected in the PVNs of MC4RF51L mice (Figure 1B). Consistent with prior results (22), α-MSH was somewhat less potent in stimulating cAMP accumulation via MC4RF51L (EC_50_: 22.6 nM [95% CI, 11.5–41.2 nM] for WT MC4R vs. 363 nM [95% CI, 219–619 nM] for MC4RF51L in HEK293^MRAP2^ cells). Whether the decreased potency was due to reduced abundance of the mutant receptor at the plasma membrane is unclear, as one study reported reduced MC4RF51L abundance at the plasma membrane (26), while another study showed no differences (22).

We next examined the ability of α-MSH to activate MC4R/G_q/11_α signaling by measuring the accumulation of inositol phosphate (IP1) in the presence of lithium, which blocks IP1 degradation, as a readout of PLC activity (Figure 7B). Similar to what we observed for cAMP accumulation, the presence of MRAP2 significantly increased the ability of α-MSH to maximally stimulate IP1 accumulation via the WT MC4R, as well as its potency. However, α-MSH was less potent for IP1 accumulation as compared with cAMP accumulation (EC_50_: 1,049 nM for IP1 vs. 22.6 nM for cAMP in HEK293^MRAP2^ cells). As compared with WT MC4R, MC4RF51L showed no IP1 response in the absence of MRAP2 and only a small increase in IP1 at high α-MSH concentrations (~10^–4^ M) in the presence of MRAP2. Accumulation of IP1 by WT MC4R in response to 10^–4^ M α-MSH was completely blocked by FR900359 (FR; formerly UBO-QIC), a specific G_q/11_α inhibitor (42–45), confirming that the IP1 responses observed in our experiments reflect effects on MC4R/G_q/11_α signaling (Figure 7E). We also observed a partial (~25%) decrease in cAMP accumulation in the presence of FR (Figure 7F). However, studies in HEK293 cells have confirmed that this effect of FR on cAMP accumulation is the direct result of G_q/11_α inhibition, rather than G_s_α inhibition (42), and other studies have found that FR does not affect G_s_α activation (43–45).

Finally, we examined MC4R/MAPK/ERK signaling by measuring the time course of ERK1/2 phosphorylation over the first 10 minutes after addition of 10^–5^ M α-MSH in HEK293^MRAP2^ cells (Figure 7, C and D). Overall, there were no statistical differences in the responses in cells expressing WT MC4R or MC4RF51L (Figure 7D). At 3 minutes the responses were similar, while at 5 and 10 minutes there were small, but statistically insignificant, decreases in responses to α-MSH in cells expressing MC4RF51L. However, FR-treated cells expressing WT MC4R showed virtually the same response as cells expressing MC4RF51L, indicating that the extent of any decrease in ERK phosphorylation observed with MC4RF51L would be due to downstream effects resulting from loss of G_q/11_α signaling. Our results showing lack of effect of the F51L mutation on MAPK signaling is consistent with prior results (28). In summary, our results show that MRAP2 is critical for both the MC4R/G_s_α and MC4R/G_q/11_α signaling pathways and that MC4RF51L has a specific defect in G_q/11_α signaling.

### MC4R-mediated food intake inhibition in PVN is blocked by G_q/11_α inhibition.

To examine the effect of G_q/11_α inhibition on the ability of intra-PVN delivery of MC4R agonist to acutely inhibit food intake, we repeated the experiment examining the ability of intra-PVN delivery of MTII to acutely inhibit food intake in WT mice, but injected FR or vehicle into the intra-PVN cannula 5 minutes prior to injection of MTII. In this experiment injection of MTII alone resulted in an approximately 70% inhibition of food intake, while FR almost completely blocked the effect of MTII on food intake (Figure 7G). Intra-PVN administration of FR alone had no effect on food intake (Figure 7H). In contrast to the effect of intra-PVN administration on food intake, intra-PVN FR injection failed to block the rise in heart rate after intra-PVN delivery of MTII (Figure 7I; compare with Figure 3E). This provides direct in vivo evidence for the role of MC4R/G_q/11_α signaling in the PVN in food intake regulation.

## Discussion

*MC4R* mutations are the most common cause of monogenic obesity and are associated with hyperphagia, increased body length, reduced energy expenditure, abnormal glucose and lipid metabolism, and decreased heart rate and blood pressure (1, 4, 35–37, 46). While some studies have suggested that cAMP in MC4R-expressing PVN neurons plays a role in the regulation of food intake and body weight (47, 48), many MC4R mutations linked to human obesity do not show defects in G_s_α/cAMP signaling (22, 23, 49), suggesting that alternative MC4R signaling pathways may also be involved in the normal maintenance of energy balance. Consistent with this notion, a study correlating the signaling properties of MC4R variants to BMI concluded that G_s_α/cAMP explains only up to 12% of the variance in BMI (25). In the present study, we further investigated the signaling properties and physiological consequences of MC4RF51L, which was reported to maintain its ability to activate the G_s_α/cAMP pathway (23). We confirmed that stimulation of CREB phosphorylation by a melanocortin agonist was maintained in the PVN of MC4RF51L mice, consistent with intact MC4R/G_s_α signaling, and that MC4RF51L maintained the ability to stimulate cAMP accumulation in response to α-MSH, with a *V_max_* similar to that of WT MC4R, although α-MSH potency was somewhat reduced.

Although MC4RF51L had relatively intact coupling to G_s_α/cAMP signaling, mice with homozygous MC4RF51L mutation developed severe obesity associated with hyperphagia and impaired anorectic responses to melanocortin agonist, as well as increased linear growth, without primary effects on cold tolerance, glucose metabolism, or cardiovascular responses to melanocortin administration. Overall the phenotypes observed in MC4RF51L mice were remarkably similar to those observed with PVNGq/11KO mice (11) (Table 2) and, along with the severe defect in G_q/11_α signaling that we observed in vitro, they implicate defective MC4R/G_q/11_α signaling in the PVN as the likely cause of hyperphagia and increased linear growth in MC4RF51L mice. Loss of Sim1, a transcription factor highly expressed in the PVN and shown to be regulated by MC4R (11), produces similar effects on food intake and linear growth and leads to loss of the anorectic response to melanocortin agonist (12–15). The major role of G_q/11_α signaling in mediating the inhibition of food intake by melanocortins in the PVN is further supported by the observation that the inhibition of food intake in response to intra-PVN delivery of MTII in WT mice was blocked by prior intra-PVN delivery of FR, a specific inhibitor of G_q/11_α. Moreover, an MC4R variant associated with protection from human obesity and lower BMI (V103I) (50–52), which has been shown to have increased ligand-induced β-arrestin recruitment (25), has also been shown to lead to increased stimulation of G_q/11_α signaling in response to α- and β-MSH (53). Finally, loss of the K^+^-dependent Na^+^/Ca^2+^ exchanger NCKX4 led to an MC4R- and Ca^2+^-dependent increase in PVN neuron activation and severe hypophagia and weight loss (54).

In contrast to what we observed in PVNGq/11KO (11) and MC4RF51L mice, G_s_α deficiency in the CNS (18) or specifically limited to the PVN (Sim1 neurons) (21) had no impact on food intake or linear growth, nor the ability of melanocortin agonist to inhibit food intake (Table 2). CNS-specific G_s_α deficiency (mBrGsKO) does lead to obesity, but this is associated with reduced energy expenditure rather than hyperphagia, and is also associated with impaired glucose metabolism, impaired cold tolerance, and reduced blood pressure (18), none of which were observed in PVNGq/11KO or MC4RF51L mice (Table 2). In contrast to mBrGsKO mice, mPVNGsKO mice do not show a significant metabolic phenotype nor impaired melanocortin action on food intake or energy expenditure, indicating that the metabolic defects observed in mBrGsKO mice do not result from loss of MC4R action in the PVN (21) (Table 2). However, like mBrGsKO mice, mPVNGsKO mice have lower blood pressure and heart rate, and intra-PVN delivery of melanocortin agonist fails to stimulate cardiovascular responses (11), consistent with G_s_α mediating the cardiovascular actions of melanocortins in the PVN. Several lines of evidence show that G_q/11_α is not involved in the cardiovascular actions of melanocortins in the PVN, including intact cardiovascular responses to intra-PVN delivery of melanocortin agonist in PVNGq/11KO mice (11) and MC4RF51L mice (the present study), as well as the inability of FR to block the cardiovascular responses to melanocortins in the PVN of WT mice (the present study).

One aspect in which the MC4RF51L mouse phenotype differed from that of PVNGq/11KO mice is that the ability of peripherally administered (i.p.) MTII to stimulate energy expenditure was absent in MC4RF51L mice, while it was maintained in PVNGq/11KO mice (Table 2). Unlike other models in which we observed the same deficit (18, 20), MC4RF51L mice did not show a reduction in baseline energy expenditure, and therefore reduced energy expenditure was not a major driver of the obesity phenotype observed under basal conditions (room temperature, ad libitum feeding). It is possible that we missed a small but significant response to i.p. MTII due to the limited numbers of mice examined. The apparent defect observed with i.p. MTII does not appear to be due to defective MC4R action in the PVN, as MTII delivery to the PVN had no effect on energy expenditure in either MC4RF51L or WT mice, consistent with the finding that reexpression of MC4R in the PVN of MC4R-null mice did not reverse the impaired ability of melanocortin agonist to stimulate energy expenditure (8). Our pair-feeding study also indicates that energy expenditure may be reduced in MC4RF51L mice when energy intake is restricted. While we have shown that the loss of G_q/11_α in the DMH leads to reduced energy expenditure, stimulation of energy expenditure in response to i.p. MTII was unaffected in these mice (55). These subtle changes in energy expenditure observed in MC4RF51L mice may reflect the loss of MC4R/G_q/11_α signaling at another site, moderately reduced ability of melanocortins to stimulate MC4R/G_s_α signaling, or impaired activation of other signaling pathways downstream of MC4R.

We performed several experiments examining the effects of the MC4RF51L mutation on downstream signaling pathways in parental HEK293 cells and HEK293 cells stably transfected with MRAP2 (HEK293^MRAP2^ cells). Consistent with prior studies, the presence of MRAP2 led to a large increase in the ability of the WT MC4R to increase cAMP accumulation in the presence of melanocortin agonist, with increases in both *V_max_* and potency (38, 56). It is unlikely that MC4RF51L has a defect in MRAP2 interaction, as the mutant receptor showed improvements in *V_max_* and agonist potency for cAMP accumulation similar to those observed for the WT receptor. WT MC4R also showed a dose-dependent increase in IP1 accumulation in response to melanocortin agonist, which was confirmed to reflect MC4R coupling to G_q/11_α, as the response was completely blocked by the G_q/11_α inhibitor FR. As was observed for the cAMP response, WT MC4R demonstrated a large increase in *V_max_* and agonist potency for the IP1 response in the presence of MRAP2. In contrast, MC4RF51L demonstrated essentially no IP1 response to agonist in the absence of MRAP2 and a minimal response at high agonist doses in the presence of MRAP2, confirming a major defect in activation of the G_q/11_α pathway. Finally, we examined MAPK activation by examining acute changes in ERK1/2 phosphorylation in response to agonist in the presence of MRAP2 and found no significant defect in the ability of MC4RF51L to stimulate ERK phosphorylation.

Recently MC4R/β-arrestin signaling has been implicated as a potentially important pathway in the regulation of body weight by a study correlating β-arrestin recruitment to MC4R variants with BMI (25). It is difficult to interpret this study, however, as β-arrestin recruitment to receptor is a general response to receptor activation, and therefore, β-arrestin recruitment may reflect the receptor being in the active conformation rather than activation of downstream β-arrestin signaling per se. The study did show a correlation between β-arrestin recruitment and ERK1/2 phosphorylation, which suggested that MAPK is activated by MC4R in a β-arrestin–dependent manner. While we did not directly measure β-arrestin recruitment to MC4RF51L, we did not see a defect in ERK1/2 phosphorylation, which might be expected if β-arrestin recruitment and/or signaling were impaired in this mutant receptor. Our study does not rule out the possibility that MC4R/β-arrestin interaction or signaling is involved in energy balance. MC4R-mediated closure of the inwardly rectifying potassium channel Kir7.1 by a G protein–independent mechanism has also recently been implicated as a regulator of energy balance and food intake (27). However, the role of Kir7.1 in body weight regulation is unclear, as Kir7.1-null mice only develop mild obesity at a very late age (57), which does not mimic the phenotype of MC4R-null (4) or MC4RF51L mice.

This study has several limitations. Although the PVN is a critical site for regulation of food intake by melanocortins, several studies have shown that the MC4R receptor is highly expressed in the dorsal vagal complex and that melanocortins delivered to this region can affect food intake and cardiovascular responses (58–60). We did not directly examine how the F51L mutation affects melanocortin actions in the dorsal vagal complex but would predict that MC4RF51L mice have altered melanocortin actions at this site that contribute to the overall phenotype. Although the results herein and in prior work (11) provide evidence that the phenotype of MC4RF51L mice is due to impaired G_q/11_α signaling, we did not rule out the possibility that other defects in MC4R function (e.g., abnormal cellular localization or protein stability; changes in potency for G_s_α/cAMP signaling; impaired β-arrestin signaling; protein-protein interactions) did not contribute to the phenotype. We also did not directly compare the phenotype of MC4RF51L mice with that of other relevant mouse lines (e.g., MC4R-null mice) in the same experiment. Finally, the results of pair-feeding experiments may be affected by the fact that the pair-fed mice do not have a normal feeding pattern, as they consume food soon after it is provided each day.

In summary, we have characterized the signaling properties and the physiological consequences of a human obesity-associated MC4R mutation and conclude that this mutation had a defect in the activation of G_q/11_α and when introduced into mice led to obesity, hyperphagia, and increased linear growth, with no significant direct effects on glucose metabolism or cardiac function, the latter of which are mediated by G_s_α (61). While melanocortin agonists have potential as a therapeutic option for obesity, its use has been limited by the presence of significant side effects, particularly increased heart rate and blood pressure and untoward sexual responses. Recent evidence shows that setmelanotide, an MC4R agonist with few reported side effects, has increased potency for activation of both G_s_α and PLC (30). Clarifying the divergent MC4R signals that mediate the diverse physiological effects of melanocortins may allow the development of more specific MC4R agonists to treat obesity. Specifically, our findings suggest that a biased MC4R agonist that preferentially activates G_q/11_α versus G_s_α may reduce food intake by increasing meal-based satiety (mediated by G_q/11_α), with lower cardiovascular side effects, which are mediated by G_s_α.

## Methods

### In vivo experiments in mice

#### Sex as a biological variable.

Our study examined male and female animals, and similar findings are reported for both sexes.

#### Generation of MC4RF51L mice.

Mice having an *Mc4r* gene mutation replacing the phenylalanine (F) at position 51 with leucine (L) (MC4RF51L mice) were generated by CRISPR/Cas9 using MC4R guide RNA with sequence CCCGAGGTGTTTGTGACTCT; repair donor DNA sequence after cutting, CCCGAGGTGTTTGTGACTCT; repair donor DNA sequence after cutting, TACGGGCTGCACGGCAATGCCAGCGAGTCGCTGGGGAAGGGCCACCCGGACGGAGGATGCTATGAGCAACTTTTTGTTTCCCCCGAGGTGCTAGTGACTCTGGGTGTCATAAGCCTGTTGGAGAACATTCTAGTGATCGTGGCGATAGCCAAGAACAAGAACCTGCACTCACCCATGTACTTT; and spCas9 mRNA (System Biosciences). Mice were maintained on a C57BL/6J background and bred to homozygosity to generate MC4RF51L mice. Mice were housed on a 12-hour light/12-hour dark cycle (with light from 0600 hours to 1800 hours) and fed a standard chow diet (NIH-07, 5% fat by weight, Envigo).

#### Body composition, food intake, energy expenditure, and physical activity measurements.

Body weight was measured weekly starting at 4 weeks of age. Body composition was measured in nonanesthetized mice using the EchoMRI 3-in-1 NMR analyzer (Echo Medical Systems). Energy expenditure, food intake, and activity levels were measured in 3-month-old mice using a 12-chamber CLAMS/Oxymax system (Columbus Instruments) after a 48-hour acclimation period, with each chamber sampled every 13 minutes. TEE was determined over a 24-hour period at 22°C, followed by a 24-hour period at 30°C. Total and ambulatory activity was determined by infrared beam interruption (Opto-Varimex Mini; Columbus Instruments). Resting energy expenditure (REE) was determined as the means of points measured when mice were not ambulating. Feeding frequency was calculated based upon the number of 13-minute intervals in which mice were eating over a 24-hour period, while meal size was calculated by the amount of food eaten per 13-minute interval when mice were eating. For energy balance analysis, body weight, body composition, and food consumption were measured every 7 days in young mice starting at 4 weeks of age. Energy expenditure was calculated based on a previously validated formula (31) that includes coefficients for change in food intake, lean mass, and fat mass over a given period, all expressed in kcal.

#### Cannula implantation into the PVN.

Male MC4RF51L and WT mice at age 2–3 months underwent unilateral stereotaxic implantation of a guide cannula (26G, Plastics One) at a position above the PVN at bregma — anterioposterior –0.8 mm, mediolateral +0.3 mm, dorsoventral –3.8 mm — using a stereotaxic apparatus. A dummy cannula was placed above the guide cannula to prevent leaks and blockage. After mice recovered at 7–10 days after surgery, the dummy cannula was replaced by an internal cannula (30G) with a 1.2 mm projection; thus, the PVN injection position was at dorsoventral –4.9 to –5.0 mm. Surgery was performed under anesthesia with isoflurane, and after surgery mice received subcutaneous injections of banamine (2.2 mg/kg; MWI) to minimize postoperative discomfort.

#### CREB phosphorylation in the PVN.

Mice were injected with the MC3R/MC4R agonist MTII (10 mg/g i.p., 4039778; Bachem) or saline (100 μL) and 45 minutes later were anesthetized and perfused with cold 4% paraformaldehyde by intracardiac injection. Brain sections (40 mm) containing the PVN were confirmed under the microscope and collected and pretreated with heat-mediated antigen retrieval by incubation in sodium citrate buffer (10 mM sodium citrate, 0.05% Tween 20, pH 6.0) at 95˚C for 30 minutes. The brain sections were then blocked in 2.5% horse serum (Vector Laboratories) plus 0.3% Triton X-100 at room temperature for 2 hours and incubated with anti-CREB (4820, Cell Signaling Technology), and their consecutive sections were incubated with anti-pCREB (Ser133, 9191; Cell Signaling Technology) in blocking solution overnight at 4˚C, followed by incubation with Alexa Fluor–conjugated secondary antibody (Alexa Fluor 555, A21428; MilliporeSigma). The sections were mounted with mounting medium with DAPI (Vector Laboratories). The fluorescent signals were captured and visualized with a fluorescence microscope (BZ-X800; Keyence). For quantification of pCREB and CREB signals, images were processed using haze reduction and black balance tools to ensure consistency between slides (2–4 sections/mouse). By use of the hybrid cell count and macro cell count tools, the integrated brightness was measured within a 200 μm diameter circle placed over the PVN on both sides of the third ventricle to ensure only signal from the PVN was counted.

#### Responses to MTII.

After stereotaxic cannula placement, mice were single-caged for 2 weeks. After 24 hours of fasting, mice received 200 nL PBS into the PVN unilaterally at 30 minutes prior to lights out, and food intake was measured over the first 3.5 hours after injection. After a 1-week recovery, the same procedure was followed, except mice received intra-PVN MTII (150 pmol/200 nL PBS). To examine the effect of FR900359 (formerly UBO-QIC) (42) on MTII-mediated inhibition of food intake, we pretreated mice with FR intra-PVN (150 pmol/150 nL) or vehicle 5 minutes prior to administration of MTII. For measurement of energy expenditure, mice were acclimated for 48 hours in an indirect calorimetric chamber. Nonanesthetized mice were injected with PBS (intra-PVN, 200 nL) prior to a 24-hour measurement period at 30°C, and then mice received 150 pmol/200 nL intra-PVN MTII after a 1-week recovery period. For food intake and energy expenditure responses to systemically delivered MTII, mice underwent the same procedures except receiving either i.p. PBS (200 μL) or MTII (200 μg for food intake; 10 μg/g for energy expenditure). To measure heart rate and blood pressure responses to intra-PVN MTII with or without FR, mice were acclimated to the BP-2000 specimen platform (Visitech) for 2 days prior to measurement. Blood pressure and heart rate were measured 5 minutes after PBS or drug administration following the same paradigm as described for food intake responses above.

#### Glucose tolerance test.

Overnight-fasted mice were given glucose (2 mg/g i.p.), and tail blood was collected before (time 0) and at the indicated times after glucose injection for blood glucose measurement using a Glucometer Contour (Bayer).

#### Cold tolerance test.

Rectal temperature was measured with a TH-5 rectal probe (Thermalet) inserted 1 cm deep. Before cold tolerance testing, mice were acclimated to experimental conditions at room temperature for a minimum of 48 hours, with daily measurement of rectal temperature. During cold tolerance tests, mice were housed singly without bedding, but provided food and water ad libitum, and exposed to 6°C conditions for 5 hours. Rectal temperature was measured before (time 0) and at the indicated times after exposure to 6°C conditions.

#### Biochemical assays.

ELISA kits were used for the measurement of serum insulin (Crystal Chem), leptin (R&D Systems), and adiponectin (ALPCO). Serum free fatty acids were measured using a kit from FUJIFILM (Healthcare Solutions), and triglyceride and cholesterol levels were measured using reagents from Thermo Fisher Scientific.

### In vitro signaling assays

#### Generation of a stable MRAP2 cell line (HEK293^MRAP2^).

The HEK293^MRAP2^ cell line was generated by Acrogenic Technologies. In brief, HEK293 cells (ATCC) were cultured in DMEM (high glucose, ATCC) supplemented with 10% FBS and 1% antibiotic-antimycotic (Thermo Fisher Scientific, Gibco) and transfected with Myc-DDK–tagged human MRAP2-expressing plasmid (RC203668, OriGene) using Lipofectamine 3000 Transfection Reagent (Thermo Fisher Scientific, Invitrogen) according to the manufacturer’s instructions. At 48 hours after transfection, cells were cultured in a selection medium containing 500 μg/mL Geneticin (Gibco), which efficiently eliminated 90% of nontransfected parental cells within 72 hours and 100% within 96 hours. The transfected cells were cultured in the selection medium for 4 weeks, and when 90% confluency was reached cells were split at a subcultivation ratio of 1:3. The exogenous expression of DDK-Myc–tagged human MRAP2 in the stable cell pool was evaluated by immunoblotting membrane fractions with mouse anti-DDK (FLAG) monoclonal antibody (1:250; TA50011-100, OriGene) and anti–α-tubulin antibody (CP06, MilliporeSigma) to control for loading. MRAP2 expression was verified with qRT-PCR (forward primer: 5′-ATTTTCTCGCCAAGGCAACG-3′, reverse primer: 5′-TGCTTCTGATGGCTTCCTGG-3′) normalized by β-actin (Supplemental Figure 3). HEK293^MRAP2^ cells were maintained in DMEM supplemented with 10% FBS and 1 mg/mL Geneticin at 37°C in 5% CO_2_.

#### Cell culture.

Parental HEK293 and HEK293^MRAP2^ cells were seeded in 96-well plates (5 × 10^4^ cells/100 μL per well) or 24-well plates (2.5 × 10^5^ cells/500 μL per well) and cultured overnight. On the experiment days, cells were transiently transfected with human WT-MC4R plasmid (cDNA Resource Center, catalog MC4R0400000) or F51L-MC4R plasmid that was generated via site-directed mutagenesis (Quintara Biosciences). All transfections were performed using Lipofectamine 3000 in Opti-MEM I reduced serum medium, according to the manufacturer’s protocol. At 24 hours after transfection, parental HEK293 and HEK293^MRAP2^ cells were treated with different concentrations of α-MSH (4008476, Bachem), ranging from 10^–8^ to 10^–4^ M, diluted in serum-free, antibiotic-free DMEM. For stimulation of intracellular cAMP levels, cells were treated with α-MSH plus 100 μM 3-isobutyl-1-methylxanthine (IBMX; I5879, MilliporeSigma) for 2 hours at 37°C in 5% CO_2_, and cAMP was measured using the DetectX Direct Cyclic AMP (cAMP) Enzyme Immunoassay Kit (K019-H1, Arbor Assays). For stimulation of intracellular IP1 production, cells were treated with α-MSH at various doses in lithium chloride–containing buffer provided in the IP-One Gq ELISA Kit (72IP1PEA, PerkinElmer Cisbio) for 1 hour at 37°C in 5% CO_2_, and IP1 was measured using the same kit. For experiments in which FR was used, cells were pretreated with 1 μM FR or DMSO vehicle for 3 minutes and then treated with α-MSH. Each treatment was performed in triplicate and repeated with 3 independent experiments.

#### ERK phosphorylation.

For measurement of α-MSH–stimulated ERK1/2 phosphorylation, HEK293^MRAP2^ cells that were transfected with WT or mutant MC4RF51L plasmid and pretreated with FR or DMSO vehicle were treated with 10^–5^ M α-MSH for 3, 5, or 10 minutes at 37°C and then were lysed in RIPA buffer (20-188, MilliporeSigma) with a protease inhibitor cocktail (cOmplete Protease Inhibitor Cocktail, Roche). After measurement of protein concentrations using the Pierce BCA protein assay kit (23225, Thermo Fisher Scientific), cell extracts were prepared using NuPAGE sample reducing agent (NP0009, Invitrogen) and NuPAGE LDS sample buffer (NP0007, Invitrogen), heated at 70°C for 10 minutes, and run on NuPAGE 4-12% Bis-Tris gels (NP0335BOX, Invitrogen); proteins were transferred onto PVDF membranes with iBlot (IB24001, Invitrogen). Blots were probed with rabbit anti–phospho-ERK1/2 polyclonal antibody (1:1,000, 9101S, Cell Signaling Technology) or rabbit anti-ERK1/2 monoclonal antibody (1:1,000, 4695, Cell Signaling Technology) at 4°C overnight. Membranes were then incubated in HRP-linked secondary antibodies (NA934V, MilliporeSigma) and developed with ECL substrate. ERK1/2 signals were analyzed as a ratio of phospho-ERK1/2 to ERK1/2 with ImageJ software (NIH).

### Statistics

Data are presented as mean ± SEM and were analyzed using Prism v7.00 (GraphPad). Statistical significance was determined using 2-tailed paired or unpaired Student’s *t* test or 1- or 2-way ANOVA, with correction for multiple comparisons when appropriate. Differences were considered significant at *P* < 0.05.

### Study approval

All animal studies were approved by the NIDDK Animal Care and Use Committee.

### Data availability

All data are available in the main text or the supplemental materials. Values for all data points in graphs are reported in the Supporting Data Values file.

## Author contributions

PJM, AZ, BAC, MC, and LSW conceived the project and directed the study with input from all authors. PJM, AZ, BAC, MC, OG, EK, and LSW were involved in design of experiments. PJM, AZ, BAC, HS, YL, MTJ, ZC, DRL, MBG, NL, MC, and OG performed the experiments. PJM, AZ, BAC, MC, OG, and LSW were involved in data analysis. PJM, AZ, MC, and LSW wrote the manuscript, and all authors were involved in review and editing of the manuscript.

## Supplementary Material

Supplemental data

Unedited blot and gel images

Supporting data values

## Figures and Tables

**Table 2:**
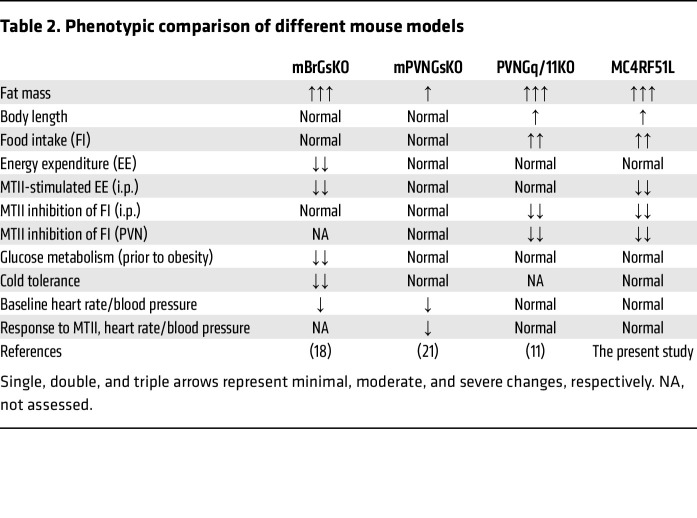
Phenotypic comparison of different mouse models

**Table 1:**
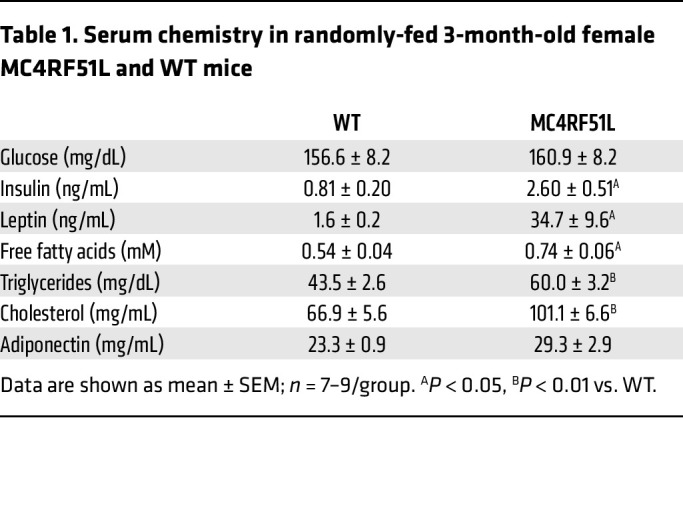
Serum chemistry in randomly-fed 3-month-old female MC4RF51L and WT mice

## Version 1 01/04/2024

In-Press Preview

## Version 2 02/15/2024

Electronic publication
