# Supplementary material for: A human obesity-associated MC4R mutation with defective Gq/11α signaling leads to hyperphagia in mice
Source: J Clin Invest. 2024 Jan 4;134(4):e165418. doi: 10.1172/JCI165418 (PMC10869179; doi:10.1172/JCI165418)
Supplement: Supplemental data [file jci-134-165418-s010.pdf]

## Supplemental Figures

A human obesity-associated MC4R mutation with defective $G_{q/11}\alpha$ signaling leads to hyperphagia in mice

Metzger, et. al.

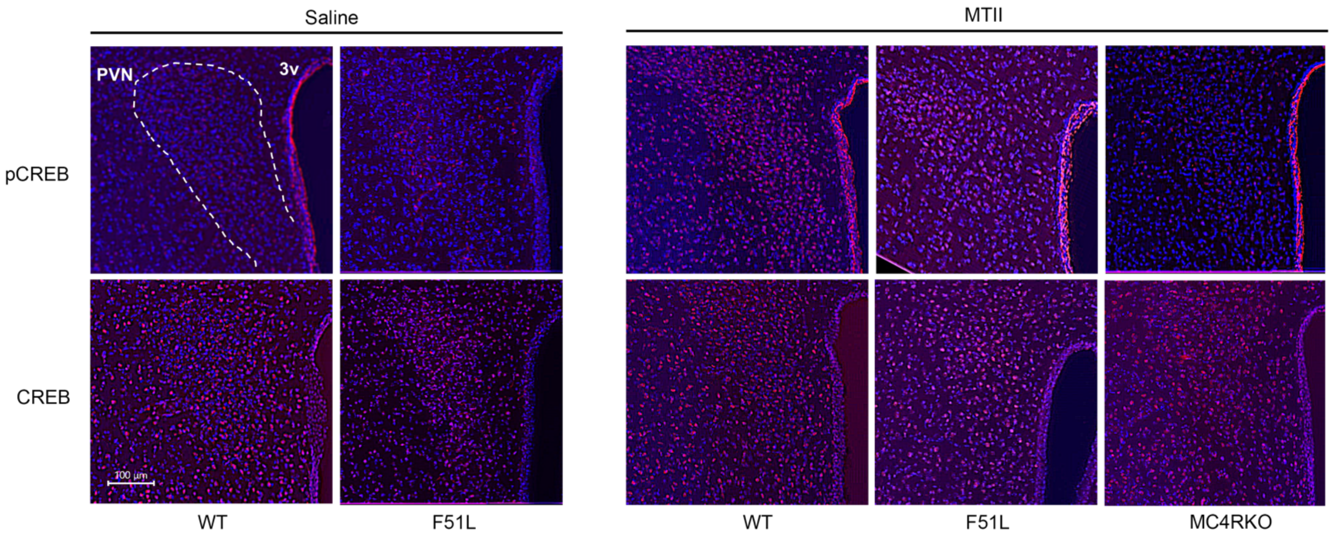


**Supplemental Figure 1. CREB phosphorylation in response to MTII.** Representative sections of PVN (outlined with dashed line) showing immunofluorescent staining using fluorescent antibodies to phosphorylated CREB (pCREB, upper row, red staining) and total CREB (lower row, red staining) in WT, MC4RF51L (F51L) and MC4RKO mice after ip. injection of either saline or MTII (scale bar = 100 µm). All slides were counterstained with DAPI.

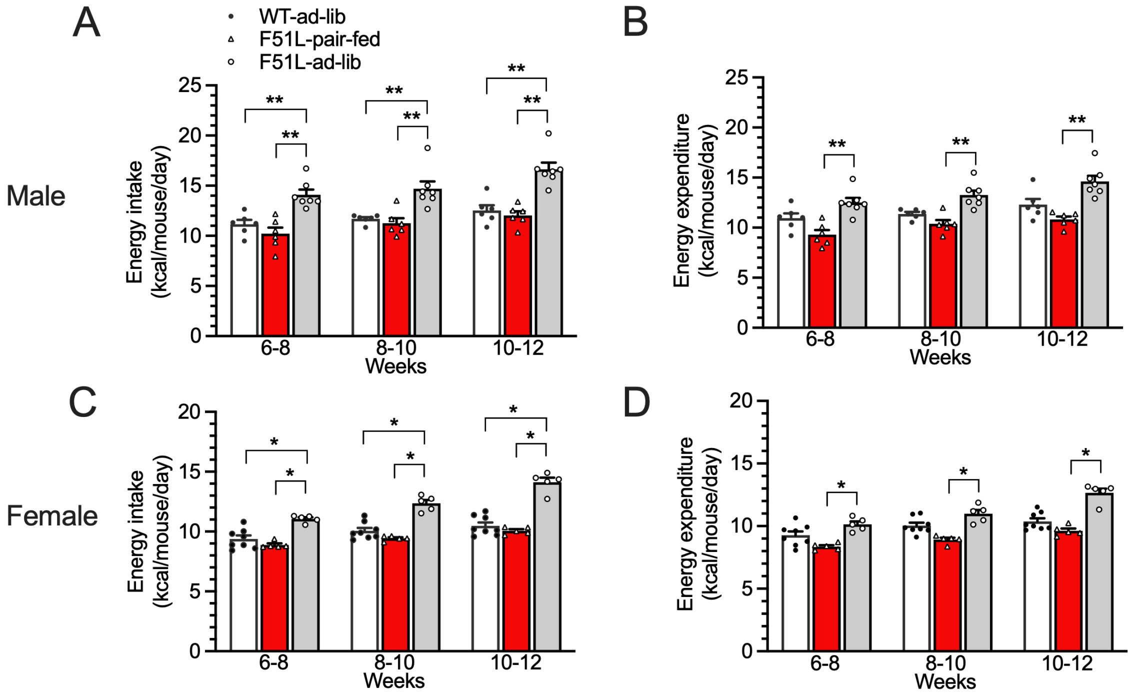


**Supplemental Figure 2. Energy balance during pair feeding study. (A-B)** Daily energy intake (**A**) and expenditure (**B**) in ad libitum-fed WT and MC4RF51L mice and pair-fed MC4RF51L mice during 2 week periods of the pair feeding (6-8, 8-10 and 10-12 weeks of age, corresponding to 2-4, 4-6 and 6-8 weeks of pair feeding, respectively; n = 6-7/group). (**C**-**D**) Energy intake (**C**) and expenditure (**D**) in female mice during pair feeding experiment (n = 5-8/group). Data represent mean ± SEM. *$p < 0.05$, **$p < 0.01$ by 1-way ANOVA.

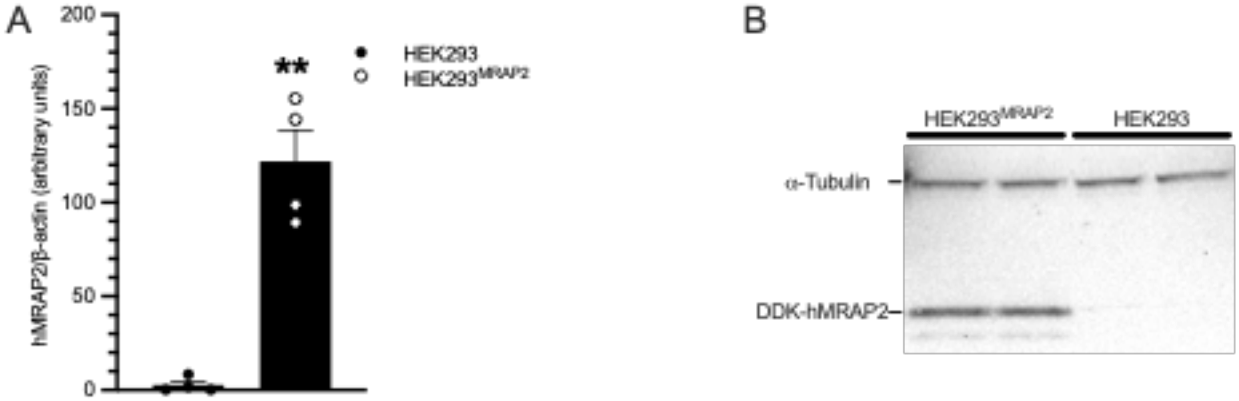


**Supplemental Figure 3. Validation of HEK293$^{MRAP2}$ cell line.** (**A**) Results of quantitative RT-PCR examining expression of hMRAP2 in parental HEK293 and HEK293$^{MRAP2}$ cells, normalized to $\beta$-actin (n = 4/group). (**B**) Immunoblot of membrane fractions from parental HEK293 and HEK293$^{MRAP2}$ cells for Myc-DDK-tagged-hMRAP2 using anti-DDK antibodies. Shown below is probing of the same blot with anti-$\alpha$-tubulin antibody. Data are expressed as mean $\pm$ SEM, **$p<0.01$ vs. parental HEK293 cells by unpaired t-test.
