# Supplementary figures and images for: A human obesity-associated MC4R mutation with defective Gq/11α signaling leads to hyperphagia in mice
Source: J Clin Invest. 2024 Jan 4;134(4):e165418. doi: 10.1172/JCI165418 (PMC10869179; doi:10.1172/JCI165418)

pERK1/2

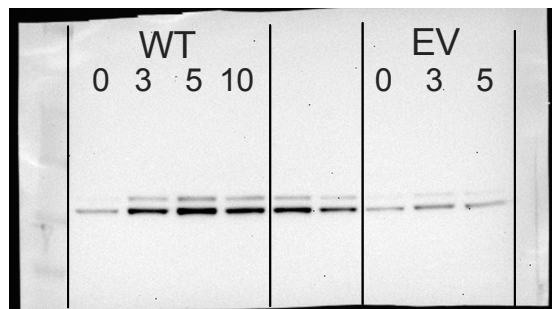

Stripped

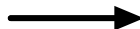

Total ERK1/2

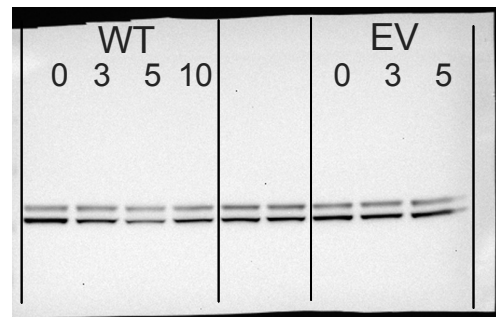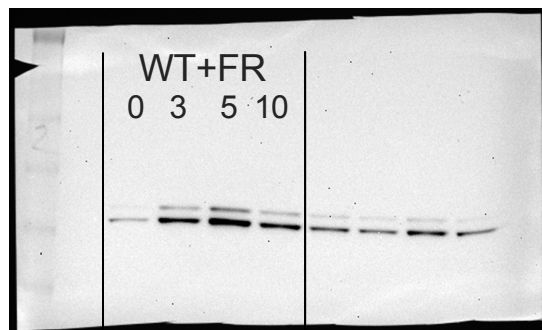

Stripped

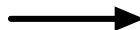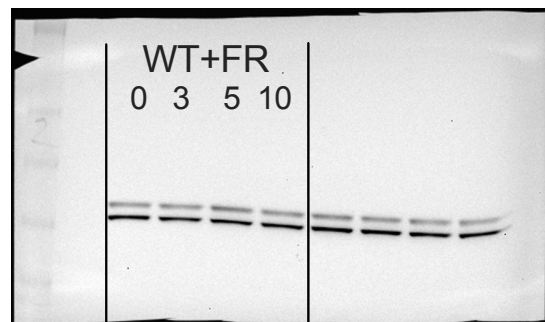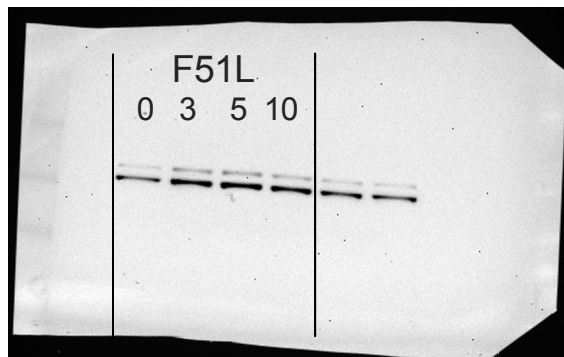

Stripped

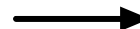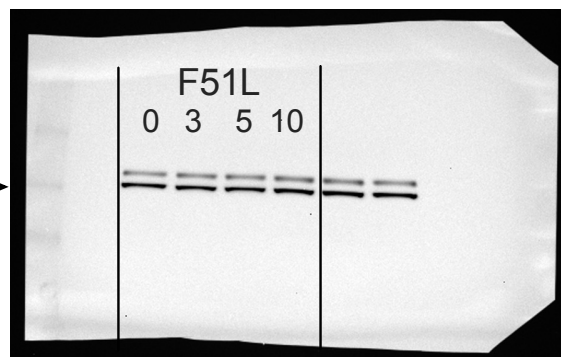

SF3B

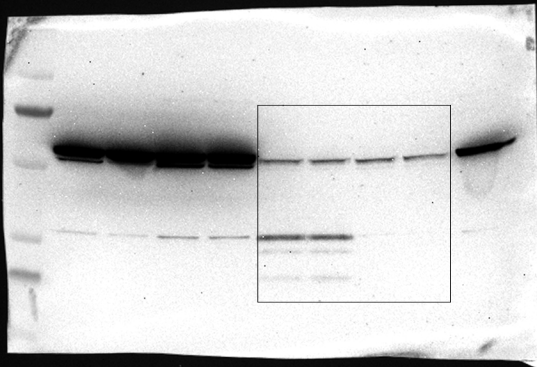

Supplement: Unedited blot and gel images [file jci-134-165418-s011.pdf]
